# Clinical and prognostic outcomes of colposcopy-guided LEEP versus cold knife conization in the management of cervical intraepithelial neoplasia

**DOI:** 10.3389/fonc.2025.1627024

**Published:** 2025-08-07

**Authors:** Fengying Jin, Lingling Wang, Zhan Su

**Affiliations:** Guang’an People’s Hospital, Gynecology Department, Guang’an, Sichuan, China

**Keywords:** colposcopy, LEEP, CIN, clinical efficacy, prognostic effect

## Abstract

**Objective:**

To evaluate the clinical effectiveness and prognostic outcomes of colposcopy-guided LEEP compared with cold knife conization (CKC) in the treatment of cervical intraepithelial neoplasia (CIN).

**Methods:**

124 patients with CIN in our hospital from January 2022 to December 2023 were chosen and classified into the control group (62 cases) and the observation group (62 cases) according to the therapeutic schedule. The observation group was treated with colposcopy combined with LEEP, while the control group underwent conventional cold knife conization (CKC). The clinical effect, surgical indicators, complications were compared. The control group underwent LEEP without colposcopic guidance, following standard clinical protocol.

**Results:**

The efficacy of the observation group was markedly better than that of the control group (95.16% vs. 75.81%, χ² = 9.358, P = 0.002). The operative time (35.35 ± 2.81 vs. 56.92 ± 2.17 minutes), intraoperative blood loss (8.08 ± 0.27 vs. 16.03 ± 2.27 mL), vaginal bleeding time (7.76 ± 1.85 vs. 11.37 ± 2.45 days), and hospital stays (2.74 ± 0.97 vs. 6.73 ± 1.33 days) were all significantly shorter in the observation group (P < 0.001). The complication rate was also lower (6.45% vs. 20.97%, P = 0.019).

**Conclusion:**

The findings suggest that this combined therapy is not only more effective but may also improve pregnancy outcomes for patients, making it a promising option for clinical application.

## 1 Introduction

Cervical cancer is one of the most common malignancies among women, ranking fourth after breast, colorectal, and lung cancers (1). In China alone, about 130,000 new cases and 50,000 deaths occur annually (2). In recent years, advancements in screening techniques and vaccination programs have been crucial in reducing cervical cancer incidence, although challenges remain in implementing these measures in resource-limited areas (3). Cervical intraepithelial neoplasia (CIN) represents premalignant changes in the cervical epithelium and is graded I–III based on severity (4, 5). High-grade lesions (CIN II–III) carry a 20–30% risk of progression to invasive cancer within 10–20 years, making early detection and treatment crucial (6). Persistent infection with high-risk human papillomavirus (HR-HPV) underlies most CIN cases, with HPV 16 and 18 accounting for roughly 70% of lesions (7–9). – Managing CIN effectively—by excising dysplastic tissue while preserving cervical function—is key to preventing cervical cancer, especially in women of reproductive age.

The therapy modalities of CIN main include cold knife cut method (CKC), loop electrosurgical excision procedure (LEEP), laser therapy and freezing therapy (15). LEEP knife surgery is a minimally invasive procedure commonly used in clinical practice, which can play a primary part in the therapy of cervical lesions. Compared with traditional surgery, it only requires local anesthesia or even no anesthesia, and does not require suture removal, which reduces the patients’ pain. Moreover, it can reduce the damage to the adjacent tissues of lesions, the intraoperative bleeding is less, the operation time is shorter (10, 11). A study on the efficacy of LEEP has demonstrated that it is effective in reducing cervical dysplasia and preserving cervical function, which is especially crucial for women of reproductive age (12, 13). However, one known drawback of the LEEP technique is the occurrence of thermal artifacts at the surgical margins, which may hinder accurate pathological assessment, particularly in determining margin status.

Colposcopy provides real-time visualization of cervical lesions and may improve the precision of LEEP procedures. While cold knife conization (CKC) remains a standard treatment for CIN, it is a more invasive procedure, requiring general anesthesia and often resulting in higher complication rates. By contrast, LEEP, especially when performed with colposcopic guidance, offers a less invasive and potentially more precise method of lesion excision. However, limited evidence is available comparing clinical outcomes between CKC with colposcopic guidance and conventional LEEP in terms of surgical success, complication rates, and fertility-related prognosis. Therefore, this study aims to evaluate the clinical and prognostic impact of colposcopy-guided LEEP compared to CKC in the treatment of CIN, particularly in populations with fertility concerns.

## 2 Materials and methods

### 2.1 General information about patients

A total of 124 patients diagnosed with CIN and admitted to our hospital between January 2022 and December 2023 were included in this study. The patients were divided into the control group (n=62) and the observation group (n=62) based on a predefined therapeutic schedule, according to the treatment protocol followed at our institution. The allocation to either the control group (cold knife conization, CKC) or the observation group (colposcopy-guided LEEP) was not influenced by clinical urgency, patient preference, or the severity of the condition.

The control group was 26–58 years old, with a mean of 35.68 ± 6.53 years. The duration of the disease ranged from 5 months to 2 years, with a mean of 1.21 ± 0.39 years. The results of physical examination were cervical smooth in 26 cases and cervical erosion in 36 cases. The CIN classification included 34 Grade II and 28 Grade III. The patients in observation group ranged in age from 25 to 56 years, with a mean of 34.78 ± 6.68 years. The duration of disease ranged from 3 months to 2 years, with a mean of 1.29 ± 0.41 years. The results of physical examination showed that 29 cases had a normal cervical appearance, while 33 cases exhibited cervical erosion. The classification of CIN was Grade II (36 cases) and Grade III (26 cases). There was no obvious difference in the general data between the two groups (P > 0.05). This study has been reviewed and approved by the hospital Ethics committee, and all patients have given their informed consent.

Sample size estimation: The minimum required sample size was calculated using a power analysis based on our previous experience, assuming a difference in total effective rate of 20% between groups, with α = 0.05 and power (1 − β) = 0.80. The calculation yielded a required sample size of at least 56 patients per group, which was exceeded in this study.

### 2.2 Inclusion and exclusion criteria

Inclusion criteria: (1) Patients who meet the diagnostic criteria for CIN in the Standardized Diagnosis and Treatment Guidelines for Cervical Cancer and Precancerous Lesions (Trial); (2) Patients confirmed by colposcopy, thin-prep cytology test (TCT), or HPV testing; (3) Patients with histologically confirmed CIN II-III on cervical biopsy; (4) Patients who are married or have a sexual history; (5) Primary patients; (6) Patients with no history of antitumor therapy; (7) Patients with good compliance and can cooperate with follow-up.

Exclusion criteria: (1) Patients with reproductive tract infection, cervical inflammation and other gynecological diseases; (2) Patients diagnosed with cervical carcinoma *in situ* or with ovarian tumors; (3) Patients with dysfunction of vital organs; (4) Patients with history of pelvic or hysterectomy; (5) Patients with coagulation and immune dysfunction; (6) Patients with mental illness or communication disorders; (7) Patients who are lactating or pregnant.

### 2.3 Methods

The observation group was treated with colposcopy combined with LEEP. The operation was performed between the 4th and 7th day after the end of menstruation. Patients were instructed to assume the bladder lithotomy position. The vulva and cervix were fully exposed after routine disinfection, and a speculum was inserted to visualize the vaginal vault and cervix. Colposcopy (OPTOMIC; Model: OP-C5) was then used to examine the cervical area. Acetic acid and iodine staining tests were performed to assess the cervical morphology and delineate the extent of lesions, which were marked accordingly. Following this assessment, local anesthesia was administered using a 1% lidocaine hydrochloride injection (Hebei Tiancheng Pharmaceutical Co., Ltd.) to facilitate the subsequent surgical procedure. And the scope and grade of lesions were further confirmed to select the appropriate LEEP cutter head (Beijing Yakokunda Medical Technology Co., LTD.) to excise the lesions. The specific operating criteria: the electric cutting power was set at 30–50 W, the incision made from 0.3 - 0.5cm away from the uncolored margin of the cervix, and the moving electrode rotated clockwise to remove the lesion in a circular manner. The excision range should exceed the lesion margin by 0.3 - 0.5mm, and the cutting depth should be determined according to the lesion grade (the depth of CIN II grade is 0.5 - 1.0 cm, the depth of CIN III grade is 1.0 - 1.5 cm). After colposcopic observation to ensure clean excision and electrocoagulation to stop bleeding, the excised lesions were sent for examination. Treatment success was defined as complete lesion excision with negative pathological margins and no visible residual lesion under follow-up colposcopy at 3 months. Postoperative care included routine cleaning, disinfection, and anti-infection treatment. Patients were advised to avoid sexual activity, vaginal medication, and douching for 4–6 weeks, and were monitored regularly during follow-up.

The control group underwent CKC. Patients were positioned and prepared similarly to those in the observation group. Using a scalpel, a cone-shaped excision was performed by making a circumferential incision at the 12 o’clock position on the cervical mucosa. The incision was directed obliquely at a 45° angle toward the cervical canal, with a depth of approximately 15 mm to ensure complete removal of the lesion. Hemostasis was achieved through direct pressure with sterile gauze.

### 2.4 Observation indicators

Evaluated based on intraoperative findings, postoperative pathology, and follow-up at 3 months after treatment. All excised specimens were sent for histopathological examination. The involvement of surgical margins (positive/negative) was recorded. Recurrence was defined as histologically confirmed CIN II or higher on follow-up.

1. Clinical effect: The evaluation was based on histological examination (biopsy). Cure was defined as complete epithelialization of the cervix and absence of residual lesions. Effective referred to ≥75% improvement in lesion healing as observed during follow-up. Cases showing less than 75% improvement were deemed ineffective. Total effective rate = (Cured cases + Effective cases)/Total cases × 100%.
2. Surgical indicators: Intraoperative blood loss, operative time, vaginal bleeding time and hospital stays were compared.
3. Complications: Postoperative complications such as vaginal bleeding, wound infection, pain and cervix adhesion were compared between the two groups.

### 2.5 Statistical methods

SPSS 27.0 analysis software was applied. All continuous variables were tested for normality before t-tests, and the Shapiro-Wilk test was used to assess whether the data conformed to normal distribution. If the data did not conform to normal distribution, nonparametric tests (such as the Mann-Whitney U test) were used for comparison. The measurement data expressed by ( $\bar{x}\pm s$ ), and t-test was adopted. The count data were statistically described by percentages, and the comparison of rates between groups was performed using the chi-square test, with P < 0.05 indicating that the difference was statistically significant.

## 3 Results

### 3.1 Results of clinical efficacy

**Figure 1:**
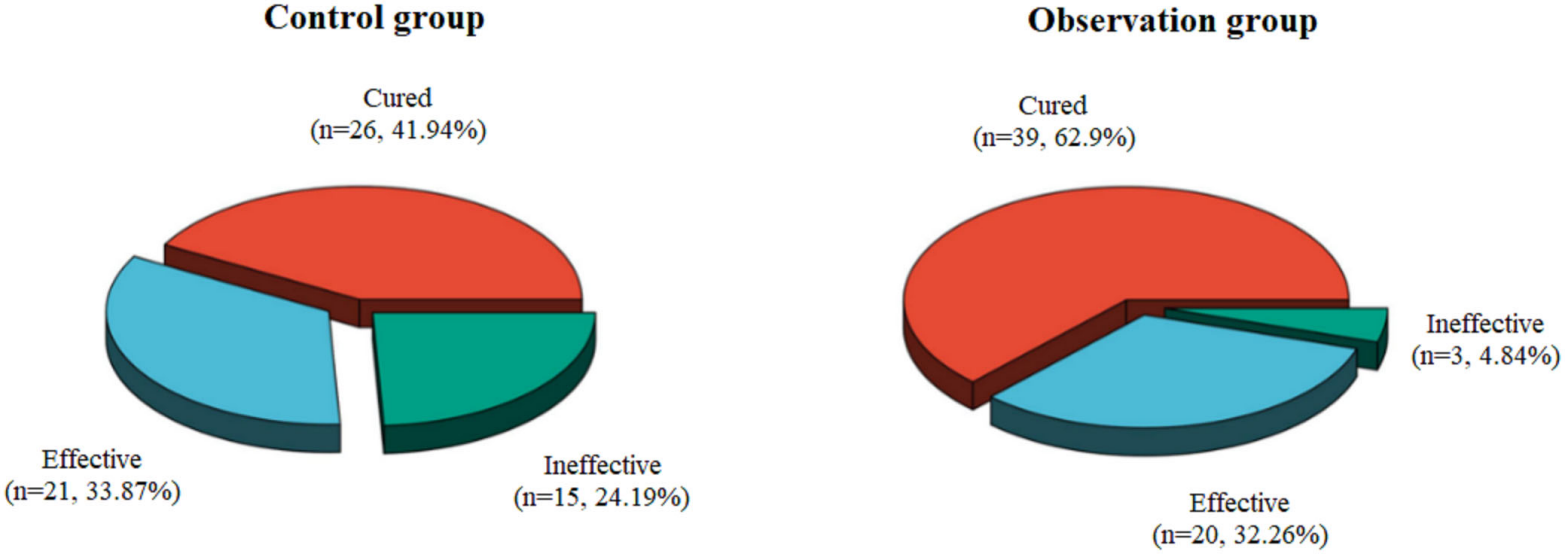
The results of clinical efficacy between the two groups.

As shown in **Table 1** and **Figure 1**, the observation group achieved a significantly higher total effective rate (95.16%) compared to the control group (75.81%) (χ² = 9.358, P = 0.002).

**Table 1:** Comparison of clinical efficacy between the two groups.

Index	Control group (n = 62)	Observation group (n = 62)	χ^2^	P
Cured [n (%)]	26 (41.94%)	39 (62.90%)	5.464	0.019
Effective [n (%)]	21 (33.87%)	20 (32.26%)	0.036	0.849
Ineffective [n (%)]	15 (24.19%)	3 (4.84%)	9.358	0.002
Total effective rate [n (%)]	47 (75.81%)	59 (95.16%)	9.358	0.002

### 3.2 Results of clinical surgical indexes

**Figure 2:**
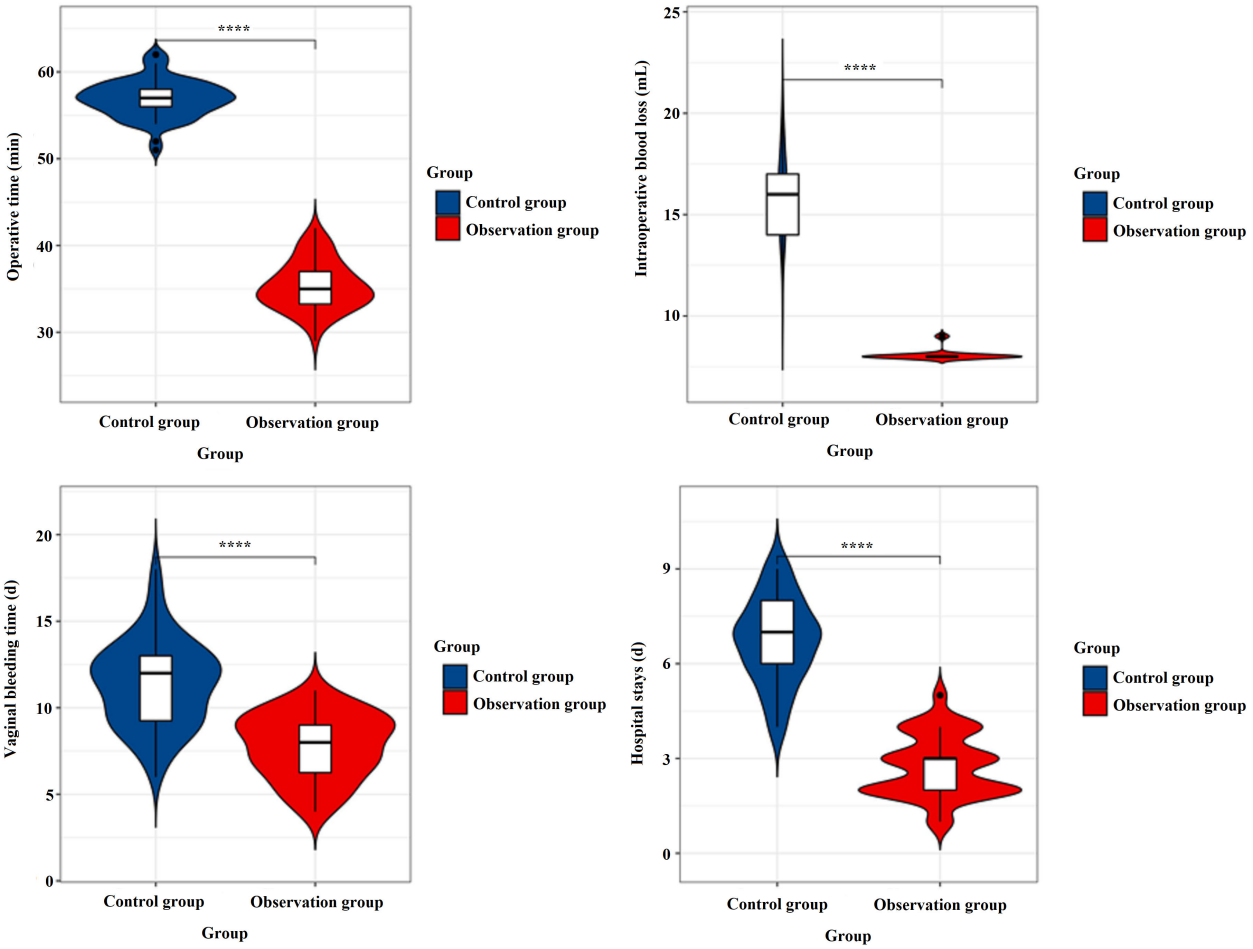
The results of clinical surgical indexes. ****: p < 0.001.

The mean operative time in the observation group was 35.35 ± 2.81 minutes, significantly shorter than 56.92 ± 2.17 minutes in the control group (t = 47.892, P < 0.001). Intraoperative blood loss was markedly reduced in the observation group (8.08 ± 0.27 mL) compared to the control group (16.03 ± 2.27 mL) (t = 27.399, P < 0.001). Vaginal bleeding time was also shorter in the observation group (7.76 ± 1.85 days) versus the control group (11.37 ± 2.45 days) (t = 9.259, P < 0.001). Additionally, hospital stays were significantly reduced in the observation group (2.74 ± 0.97 days) compared to the control group (6.73 ± 1.33 days) (t = 19.004, P < 0.001). As shown in **Table 2** and **Figure 2**.

**Table 2:** Comparison of clinical surgical indexes between the two groups.

Index	Control group (n = 62)	Observation group (n = 62)	t	P
Operative time (min)	56.92 ± 2.17	35.35 ± 2.81	47.892	< 0.001
Intraoperative blood loss (mL)	16.03 ± 2.27	8.08 ± 0.27	27.399	< 0.001
Vaginal bleeding time (d)	11.37 ± 2.45	7.76 ± 1.85	9.259	< 0.001
Hospital stays (d)	6.73 ± 1.33	2.74 ± 0.97	19.004	< 0.001

### 3.3 Results of the occurrence of complications

As shown in **Table 3**, the total complication rate in the observation group was significantly lower than in the control group (6.45% vs. 20.97%, χ² = 5.522, P = 0.019). Specifically, the rates of vaginal bleeding (1.61% vs. 3.23%), wound infection (3.23% vs. 6.45%), pain (1.61% vs. 6.45%), and cervix adhesion (0% vs. 4.84%) were all lower in the observation group compared to the control group.

**Table 3:** Comparison of complication rate between the two groups.

Index	Control group (n = 62)	Observation group (n = 62)	χ^2^	P
Vaginal bleeding [n (%)]	2 (3.23%)	1 (1.61%)		
Wound infection [n (%)]	4 (6.45%)	2 (3.23%)		
Pain [n (%)]	4 (6.45%)	1 (1.61%)		
Cervix adhesion [n (%)]	3 (4.84%)	0 (%)		
Total occurrence rate [n (%)]	13 (20.97%)	4 (6.45%)	5.522	0.019

## 4 Discussion

The occurrence and progression of cervical cancer is thought to be a multi-factor, multi-step, multi-stage dynamic development of the pathological process, which is from normal cervical-HPV infection to CIN to cervical cancer progression, which is a relatively long process, about ten years or even decades (14, 15). CIN is the precancerous stage of cervical cancer, the prevalence of CIN I resolves naturally, only a small percentage of lesions persist or develop into CIN II - CIN III, and roughly 10 - 20% of CIN II - CIN III ultimately develop into cervical cancer (16). Since it is necessary to go through a relatively long stage of precancer to develop cervical cancer, timely diagnosis and correct therapy of patients at the stage of precancer are likely to potentially reduce the occurrence and mortality for cervical cancer. Recently, the occurrence of CIN has shown an obvious trend of rejuvenation, and more CIN patients require the preservation of reproductive function (17, 18). Therefore, it is particularly vital to maximize the preservation of the reproductive potential of young patients with CIN. In recent years, LEEP has become the main therapy method for CIN. It is a therapeutic means of conical excision of suspected diseased cervical tissues by using an electric knife with a circular or triangular metal ring, which is passed through a high-frequency electric current (19). Through the high-frequency current can quickly generate ultra-high frequency radio waves, in contact with the human soft tissue that can generate impedance to generate a large amount of heat energy, so that the cells instantly become explosive dehydrated tissue. The various operations such as cutting and hemostasis are accomplished without affecting the pathological examination of the organisms at the margins of the incision (20, 21).

The mechanism of LEEP is the principle of high heat, which can improve the surgical accuracy and reduce the damage to normal tissue (22). At the same time, the operation can preserve the fertility function and meet the fertility needs of patients. In our study, colposcopy combined with LEEP was used to make full use of the role of colposcopy, which could improve the surgical field of view, promote the grasp of the disease information, and maximize the removal of diseased tissue, thus improving the clinical effect (23–25). Our study revealed that the efficacy of the observation group was better than the control group (P < 0.05), indicating that the combined application of colposcopy and LEEP can enhance the therapeutic effect and promote clinical efficacy, and has good application value. Because colposcopy combined with LEEP can achieve complementary advantages, colposcopy provides a good operating field, combined with arc resection can achieve complete resection of lesions. It is conducive to hemostasis, so as to effectively improve clinical therapy effect (26). At the same time, our results indicated the operative time, vaginal bleeding time, intraoperative blood loss and hospital stays in the observation group were lower (P < 0.001), suggesting that colposcopy combined with LEEP could shorten the operation time and discharge time, reduce the amount of intraoperative blood loss. The reason may be that the use of colposcopy can more clearly observe the cervical lesions, especially the relationship between the lesion and the normal tissue, so as to promote the complete removal of the lesion (27). Meanwhile, a good surgical field is conducive to the refinement and accurate operation of the operation, thus effectively shortening the operation time, reducing the trauma to the patient, and providing favorable conditions for a good postoperative recovery (28).

Our results presented the occurrence of complications in the observation group was markedly reduced (P < 0.05), indicating that the above combined therapy regimen could prevent complications, not only improve the safety of LEEP, but also promote a good prognosis. We analyzed that the combined application of colposcopy and LEEP could maximize the resection of the lesion and facilitate the observation of normal tissues around the lesion, thus reducing unnecessary damage and preventing the occurrence of cervical adhesion and bleeding (29, 30). “In addition to the immediate clinical outcomes, the long-term recurrence of HPV-related lesions remains a key concern in the management of CIN. Several studies have explored the long-term risk factors for recurrence after surgical interventions such as conization. For example, a retrospective multi-institutional study highlighted the role of HPV vaccination in reducing the recurrence risk of cervical dysplasia after conization (31). Although the study did not find a statistically significant reduction in recurrence, it suggested that vaccination slightly lowers the risk of recurrent disease, particularly in patients who had at least one negative examination between conization and the diagnosis of recurrent lesions. This finding underscores the importance of integrating vaccination strategies in cervical dysplasia management to potentially reduce the risk of recurrence over time. Thus, while colposcopy-guided LEEP is effective in the short term, additional interventions, including vaccination, should be considered to further reduce the risk of recurrence and improve long-term patient prognosis. It is essential for future research to assess the cost-effectiveness and broader application of HPV vaccination, particularly in women undergoing conization for CIN”.

This study has several limitations. As a retrospective, single-center design, it is subject to selection bias and observer bias, which may affect the generalizability of the results. The small sample size further limits the external applicability of our findings. The lack of randomization in this study, with allocation based on therapeutic schedule, could have introduced selection bias. The lack of colposcopy in the control group may have introduced detection bias. Moreover, the follow-up period for lesion recurrence was limited to only 3 months, which may not be sufficient for assessing long-term CIN outcomes. While we controlled for some confounding factors, unmeasured variables could still influence outcomes. To confirm our findings, prospective, multicenter, and larger-scale studies with randomized designs are needed to minimize biases and improve the reliability of the results.

## 5 Conclusion

In summary, colposcopy combined with LEEP is an effective therapy for high-grade CIN, particularly CIN II and CIN III. This approach can improve clinical outcomes, reduce intraoperative blood loss, shorten operation time, and minimize hospital stays. These advantages highlight the potential of colposcopy combined with LEEP as a valuable treatment option for high-grade CIN. However, the retrospective nature, single-center design, and small sample size of this study limit the generalizability of the findings.

## Data Availability

The raw data supporting the conclusions of this article will be made available by the authors, without undue reservation.
